# Effects of dietary palygorskite supplementation on the growth performance, oxidative status, immune function, intestinal barrier and cecal microbial community of broilers

**DOI:** 10.3389/fmicb.2022.985784

**Published:** 2022-08-25

**Authors:** Mingfang Du, Yueping Chen, Shiqi Wang, Haoran Zhao, Chao Wen, Yanmin Zhou

**Affiliations:** College of Animal Science and Technology, Nanjing Agricultural University, Nanjing, China

**Keywords:** palygorskite, antibiotic, broilers, immune function, oxidative status, intestinal barrier, cecal microbial community

## Abstract

The present study aimed to investigate the effects of palygorskite (PAL) as an alternative to antibiotic on the growth performance, oxidative status, immune function, intestinal barrier and cecal microbial community of broilers. A total of 360 1-day-old male Ross-308 broilers were randomly allotted to three treatments with eight replicates. Broilers in the three groups were designated as follows: basal diet (CON group), basal diet+50 mg/kg chlorotetracycline (ANT group), and basal diet+ 10 g/kg PAL (PAL group). Supplementing PAL reduced feed to gain ratio in broilers during 22 to 42 days of age (*P* < 0.05), with its value being similar to that of the ANT group (*P* > 0.05). Broilers fed a PAL-supplemented diet exerted decreased contents of interferon-γ (IFN-γ) and interleukin-1β in serum, and the same reduction was found in jejunal IFN-γ level, when compared to the CON group (*P* < 0.05). Moreover, compared with the CON group, broilers after PAL treatment had a lower malondialdehyde content in jejunal mucosa (*P* < 0.05). Supplementing PAL elevated jejunal villus height (VH) and ratio of VH to crypt depth compared with the ANT group (*P* < 0.05). Cecal microbiota communities among the three groups were significant different, as demonstrated by distinct clusters from partial least squares discriminant analysis, although dietary treatments had no significant effects on the bacterial richness and diversity indices (*P* > 0.05). At genus level, the addition of PAL increased the relative abundance of *norank_f__Barnesiellaceae* and decreased that of *unclassified_f__Oscillospiraceae* in cecal digesta compared with those in the CON group (*P* < 0.05); the proportion of genus *norank_f__Barnesiellaceae* was increased by PAL treatment when compared with the ANT group (*P* < 0.05). Moreover, spearman's correlations showed that the modulation of cecal microflora composition by PAL supplementation was closely correlated with the promotion of growth performance (feed to gain ratio) and intestinal health-related (contents of malondialdehyde and IFN-γ, and VH value in jejunum) variables of broilers (*P* < 0.05). Taken together, dietary PAL could improve the growth performance, antioxidant capacity, and immune status, as well as intestinal barrier function in broilers, which might be partially associated with the alteration of cecal microbiota. Moreover, dietary PAL may be a promising alternative to antibiotic growth promoter for broilers.

## Introduction

In the 1940s, antibiotic additive in feed was proven to have favorable effects in productive performance in animals (Dibner and Richards, 2005). From then on, the administration of antibiotics in feed as antibiotic growth promoters (AGPs) has become a common practice for non-medicinal purpose (Bacanli and Başaran, 2019). The AGPs can improve growth performance, feeding efficiency and economic benefits through the modulation of the intestinal microbiota and the prevention of pathogenic infection (Robinson et al., 2019). However, the long-term use of AGPs has induced antibiotic drug pollution and aggravated antimicrobial resistance in pathogenic bacteria, both of which have seriously threatened the global public health (McEvoy, 2002; Menkem et al., 2019). Due to these severe security concerns, many countries and regions have implemented strict legislations in order to eliminate the usage of antibiotics for their sub-therapeutic use (Manyi-Loh et al., 2018; Bacanli and Başaran, 2019). China has prohibited the use of antibiotic as growth promoters for the purpose of animal health maintenance since 1 July 2020 (Ministry of Agriculture and Rural Affairs, 2019). Intestinal health is of particular importance for the health, welfare and performance of broiler chicken (Roberts et al., 2015). However, the intestinal tract of broilers is susceptible to exterior stressors, especially under intensive rearing systems (Ducatelle et al., 2018). Scientific evidences suggested that the withdrawal of AGPs has increased prevalence of enteric diseases which were previously controlled by antibiotic (e.g., subclinical necrotic enteritis), and ultimately compromised the growth performance of broilers (Ducatelle et al., 2018; Khalique et al., 2020). Therefore, there is an urgent need to develop novel alternatives for maintaining intestinal health of broilers with antibiotic-free diet.

Clay minerals have been considered as promising alternatives to AGPs, due to their specific structures, as well as special physical and chemical properties (Gadde et al., 2017). Some clay minerals were proposed and tested in animals to improve health status and production performance, such as bentonite (Attar et al., 2018), montmorillonite (Liu H. et al., 2020) and zeolite (Qu et al., 2019). Palygorskite (PAL), also known as attapulgite, is a kind of hydrated magnesium aluminum silicate with ribbons of 2:1 phyllosilicate unit, possessing rod-shaped crystals, plentiful pores, and active silanol groups, which in turn endow it with particular characteristics (Murray, 2000; McKeown et al., 2002). PAL has garnered an increased interest for its application in animal nutrition. The fundamental properties for the wide application of PAL in feedstuffs are its large surface area, strong adsorption function, great exchangeability, and eco-friendly feature (Zhang et al., 2015, 2020). Several studies performed on poultry have showed that the administration of PAL exerted positively effects on the growth performance (Wang et al., 2019), digestive function (Qiao et al., 2015), intestinal health (Chen et al., 2016), and product quality and safety (Cheng et al., 2018). In broilers, PAL has been widely adopted as either feed additive or feed raw material, and its optimal dosage is 10 g/kg (Zhang et al., 2017). Dietary supplementation with PAL could beneficially improve intestinal antioxidant status, immunity, ultimately resulting in the enhanced intestinal barrier function of broilers (Chen et al., 2016). Moreover, PAL has a high *in vitro* adsorption or adhesion for bacteria (Hui et al., 2020). Meanwhile, the nanorods of PAL may cause “needle stab effect” and subsequently destroy the cytoplasmic membrane to inhibit the growth of bacteria (Hui et al., 2020; Yang and Wang, 2022). Moreover, previous studies have demonstrated that dietary PAL was effective to modulate cecal microbial community *in vivo* (Zhang et al., 2013; Chalvatzi et al., 2016; Jin et al., 2021). It is generally known that cecal bacteria have a major impact on intestinal function and animal health. Based on the results of previous studies, we supposed that PAL supplementation could serve as a substitute to APGs and the benefits of PAL on broilers may be followed by the regulatory effect of cecal bacterial flora. In the current study, PAL was used as an antibiotic alternative. Moreover, the objective of this trial was to investigate the effects of PAL supplementation on the oxidative status, immune response and intestinal barrier function, and further explore the correlation of cecal microbiota composition with growth performance and intestinal health in broilers.

## Materials and methods

### Palygorskite

The supplemented PAL was provided by the Xuyi Oubaite Clay Materials Co., Ltd (Huaian, China). It was smashed as powder to pass through a 200-mesh sieve prior to further use. The PAL sample was examined using a Zetasizer Nano ZS analyzer, Model ZEN3600 (Malvern Instruments Ltd., Malvern, UK) and presented a mean particle size of 114.5 μm. The main chemical component of PAL was determined by an Epsilon 3 X-ray fluorescence (XRF) spectrometer of PANalytical (Almelo, Netherlands), including SiO_2_, 59.70%; Al_2_O_3_, 14.66%; Fe_2_O_3_, 6.90%, CaO, 3.42%; MgO 2.74; K_2_O, 1.92%; Na_2_O, 0.30%.

### Animal treatment and experiment design

A total of 360 1-day-old male Ross-308 broiler chicks with similar initial body weight (44.05 ± 0.12 g) were obtained from commercial hatchery. The chicks were randomly allotted into three treatments with eight replicates per treatment and 15 birds per replicate. Broilers in the three treatments were fed the following diets for the 42-day feeding trial: (1) basal diet (CON group); (2) basal diet + 50 mg/kg chlorotetracycline (by effective content, ANT group); (3) basal diet + 10 g/kg PAL (PAL group). Two basal corn-soybean meal diets were formulated according to the nutrient requirements for 1–21-day starter and 22–42-day grower broilers, respectively, as declared by The National Research Council (1994), and their ingredient composition and calculated nutrient level were shown in Table 1. Chicks were housed in the 3-level-wired cages in a temperature-controlled house. The temperature was kept at approximately 33°C for the initial 3 days and gradually reduced to 24°C with the relative humidity maintaining at 60 to 70% during the experimental period. Mash feed and water were provided *ad libitum* and lighting program was kept on a cycle of 23-h light and 1-h darkness throughout the 42 days feeding trial. Vaccination protocol was implemented according an immunization program for broiler chickens as described by Chen et al. (2020b).

**Table 1:** Ingredient composition and calculated nutrition content of basal diet for Ross-308 broilers (g/kg, as-fed basis unless otherwise stated).

**Items**	**Starter**	**Grower**
	**1–21 days**	**22–42 days**
**Ingredients**		
Corn	570	620
Soybean meal	326	280
Corn protein meal	30	20
Soybean oil	30	40
Dicalcium phosphate	20	16
Limestone	12.3	13
L-Lysine	3.2	3.1
DL-Methionine	1.5	1.1
Sodium chloride	3	3
Premix^1^	4	3.8
Total	1,000	1,000
**Calculated nutrient levels**		
Metabolizable energy (MJ/kg)	12.55	12.98
Crude protein	215.5	193.3
Lysine	12.2	11
Methionine	5	4.3
Methionine + cystine	9	7.2
Calcium	10.1	9.3
Available phosphorus	4.6	3.9

^1^Premix provided per kilogram of diet: Vitamin A (transretinyl acetate), 12,000 IU; Vitamin D_3_ (cholecalciferol), 3,000 IU; Vitamin E (all-rac-α-tocopherol acetate), 30 IU; menadione, 1.3 mg; thiamin, 2.2 mg; riboflavin, 8 mg; Vitamin B_12_ (cobalamin), 0.013 mg; nicotinamide, 40 mg; choline chloride, 400 mg; D-Calcium pantothenate, 10 mg; pyridoxine·HCl, 4 mg; biotin, 0.04 mg; folic acid, 1 mg; Fe (from ferrous sulfate), 80 mg; Cu (from copper sulfate), 7.5 mg; Mn (from manganese sulfate), 110 mg; Zn (from zinc oxide), 65 mg; I (from calcium iodate), 1.1 mg; Se (form sodium selenite), 0.3 mg.

### Determination of growth performance

The average initial body weight was recorded for each replicate at 1 day of age. At 21 and 42 days of age, broilers were weighed by replicate after a 12-h feed withdrawal to calculate average daily gain (ADG). The feed intakes per replicate in starter period (1 to 21 days of age) and grower period (22 to 42 days of age) were recorded to determine the average daily feed intake (ADFI). The mortality was also registered to calculate the feed to gain ratio (F/G) during the experimental period.

### Sample collection

On days of 42, one 12-h-fasted bird was randomly selected from each replicate (eight birds in each experimental group), respectively. Blood specimens were taken from the wing vein and collected in anticoagulant-free plastic tubes and pyrogen-free glass tubes coated heparin sodium-anticoagulant to obtain serum and plasma, respectively. Serum samples were isolated from plastic tubes by centrifugation at 4°C, 4,000 *g* for 15 min. Plasma samples were separated from glass tubes after centrifugation at 4°C, 3,000 *g* for 3 min. Serum and plasma were stored at −20°C for further analysis. Broilers were then euthanized by cervical dislocation and necropsied immediately. The jejunum tissues (from pancreas-biliary ducts to the Meckel's diverticulum) were removed from the euthanized birds. Segments of mid-jejunum (~2 cm) were excised, flushed gently twice with ice-cold phosphate buffer, and fixed rapidly in 4% paraformaldehyde solution for morphological examination. The rest of jejunum samples were opened longitudinally and cleaned thoroughly with ice-cold physiological saline. The jejunal mucosa was scraped carefully, placed in cryogenic vials, and stored directly in liquid nitrogen until subsequent analysis. The cecal luminal content was aseptically collected in sterile tubes, and frozen in liquid nitrogen for later DNA extraction.

### Preparation of mucosal homogenate

Jejunal mucosa samples were diluted in 1:4 (wt/vol) with ice-cold 154 mmol/L sodium chloride solution, and homogenized in an ice-water bath using a Bio-Gen PRO200 homogenizer (PRO Scientific Inc., Oxford, Connecticut, USA) until no tissue particles were visible in the solution. The mucosal homogenate was centrifugated at 4,450 *g* for 15 min at 4°C. The supernatant was stored at −20°C for the determination of the antioxidant-related and immune parameters. The total protein content in the acquired supernatant was measured with the bicinchoninic acid colorimetric assay as previously described by Smith et al. (1985).

### Measurement of antioxidant-related parameters

To evaluate the antioxidant status of broilers, activities of glutathione peroxidase (GPX, No. A005-1-2) and superoxide dismutase (SOD, No. A001-1-2), level of total antioxidant capacity (T-AOC, No. A015-1-2), as well as content of malondialdehyde (MDA, No. A003-1-2) in serum and mucosal supernatant were determined. All the samples were diluted to the appropriate concentration after defrosting and measured using commercial diagnostic kits purchased from Nanjing Jiancheng Bioengineering Institute (Nanjing, China) according to the manufacturer's recommended procedures. In brief, GPX and SOD activities were analyzed with 5,5'-dithiobis (2-nitrobenzoic acid) method and hydroxylamine method, T-AOC level was quantified by the morphine complex with ferrous ions, and MDA content was determined by thiobarbituric acid method, separately. Absorbance values were detected using a Spark multimode microplate reader (Tecan Group Ltd., Maennedorf, Switzerland). The results of antioxidant-related parameters in jejunal mucosa were normalized against corresponding total protein level for inter-sample comparison.

### Assay of immune indices

The enzyme linked immunosorbent assay (ELISA) was performed to examine the concentrations of immunoglobulins and inflammatory cytokines in both serum and jejunal mucosa following the protocols of instructions. The ELISA quantitation kits were purchased from Nanjing Jiancheng Bioengineering Institute (Nanjing, China), including immunoglobulin A (IgA, No.H108), tumor necrosis factor-α (TNF-α, No. H052), interferon-γ (IFN-γ, No. H025) and interleukin-1β (IL-1β, No. H002). In detail, flat-bottom microtiter plate pre-coated with corresponding chicken-specific antibody was incubated under room temperature for 30 min before the determination. Wells were blocked with standards or samples to combine with the antibody, followed by horseradish peroxidase (HRP)-conjugated antibody specific for target enzyme. After incubation which was performed at 37°C for 1 h, plates were washed five times with washing buffer. Wells that contained enzyme and HRP-conjugated enzyme antibody appeared blue in color under the catalysis of the tetramethylbenzidine substrate. The color reaction was stopped by the diluted sulfuric acid stop solution, and turned into yellow. The absorbance at 450 nm denoted negatively the antigen density of sample. The results of jejunal mucosa were corrected by total protein concentration as aforementioned described.

### Examination of intestinal morphology

The paraformaldehyde-fixed jejunal sections were dehydrated using ethyl alcohols of increasing-gradient concentration, cleared in xylene, permeated with paraffin, and finally embedded in paraffin blocks *via* a KD-BM tissue embedder (Zhejiang Jinhua Kedi Instrumental Equipment Co., Ltd., Jinhua, China). Several cross-sections were sliced at a thickness of 5 μm, mounted on glass slides, deparaffinized in xylene, rehydrated, and stained with hematoxylin and eosin. Slides were analyzed morphologically on 8 villi chosen from each segment under a Nikon Eclipse 80i light microscope (Nikon Corporation, Tokyo, Japan) equipped with NIS-Elements 3.0 Imaging Software. The intestinal morphology examination included the following variables: villus height (VH, from the tip of the villus to the crypt), crypt depth (CD, from the base of the villi to the submucosa), and the villus height to crypt depth ratio (VH/CD).

### Determination of plasma biomarkers of intestinal permeability

D-lactate (D-LA), and lipopolysaccharide (LPS) in plasma are reliable parameters to assess the intestinal permeability. Levels of D-LA (No. H263) and LPS (No. H255) in plasma were assayed using an ELISA technique as above mentioned.

### DNA extraction and illumina MiSeq sequencing

Total bacterial genomic DNA from cecal content was extracted *via* the E.Z.N.A.^®^ soil DNA Kit (No. D5625-02, Omega Bio-Tek, Norcross, USA) in the light of the manufacturer's instructions. The concentration and quality of DNA extract were checked by nano-spectrophotometer (Thermo Scientific, Wilmington, USA) and 1% agarose gels. V3–V4 regions of the bacterial 16S rRNA gene were amplified with the primer pairs 338F (5'-ACTCCTACGGGAGGCAGCAG-3') and 806R (5'-GGACTACHVGGGTWTCTAAT-3'). After amplification, PCR products were identified by 2% agarose gel, and refined with AxyPrep DNA Gel Extraction Kit (No. AP-GX-50G, Axygen Biosciences, Union City, USA). The purified amplicons were pooled in equal amount, and paired-end sequenced on an Illumina MiSeq PE300 platform (Illumina Inc, San Diego, USA) at Shanghai Majorbio Bio-Pharm Technology Co., Ltd. (Shanghai, China).

### Microbiota characterization

The raw 16S rRNA gene sequencing reads were demultiplexed, quality filtered by fastp version 0.20.0, and merged by FLASH version 1.2.7. The sequence reads were clustered into operational taxonomic units (OTUs) with a 97% similarity using UPARSE version 7.1, and chimeric sequences were identified and removed. The taxonomy of each OTU representative sequence was analyzed using the RDP Classifier version 2.2 against the 16S rRNA database (Silva v138) with a confidence threshold of 0.7. The obtained high-quality data was used for the subsequent analysis with the online platform of Majorbio Cloud Platform (www.majorbio.com).

Venn diagram was generated to visually show the common and unique OTUs among groups, regardless of their relative abundances. Rarefaction curves were constructed to evaluate the sample efficiency, and prevent methodological artifacts arising from varying sequencing depths. The α-diversity indices were estimated, including observed richness (Sobs) for community richness, Shannon diversity index (Shannon) for community diversity, and Good's coverage for the community coverage. Bacterial β-diversity was compared by principal coordinates analysis (PCoA), partial least squares discriminant analysis (PLS-DA), and similarity analysis (ANOMIS) with bray-curtis distance. Taxonomic compositions at the phylum and genus levels were investigated and displayed with a bar map.

### Statistical analysis

Data analysis was done using one-way analysis of variance (ANOVA) followed by Duncan's test of SPSS version 19.0 software. The tabular data were shown as mean, standard error of the means, and *P* values. Results with *P* < 0.05 were considered as a significant difference. Spearman's correlation analysis of cecal bacterial community components with growth performance and intestinal health-related variables were determined and represented with a heatmap by R version 3.3.1. Figures were performed on the Majorbio Cloud Platform and GraphPad Prism version 8.0.

## Results

### Growth performance

The growth performance of broilers from different dietary treatments was shown in Table 2. Compared with CON group, the addition of PAL and ANT to the feed reduced F/G during 22–42 days of age (*P* < 0.05). Moreover, birds in ANT treatment had lower F/G during the whole experimental period (1 to 42 days) when compared with CON group (*P* < 0.05), with the value of the parameter being similar to that of PAL-supplemented group (*P* > 0.05). However, no significant changes were observed for the ADG and ADFI among groups during starter, grower and whole periods (*P* > 0.05).

**Table 2:** Effects of dietary palygorskite supplementation on the growth performance in broilers.

**Items^1, 2^**	**CON**	**ANT**	**PAL**	**SEM**	***P*-value**
**1–21 day**					
ADG (g/day)	32.28	31.90	32.67	0.23	0.425
ADFI (g/day)	44.32	44.31	45.32	0.35	0.423
F/G (g/g)	1.37	1.39	1.39	0.01	0.319
**22–42 day**					
ADG (g/day)	76.60	79.70	76.91	0.75	0.181
ADFI (g/day)	145.65	143.62	141.00	1.45	0.441
F/G (g/g)	1.90^a^	1.80^b^	1.83^b^	0.02	0.021
**1–42 day**					
ADG (g/day)	55.64	57.35	55.97	0.43	0.231
ADFI (g/day)	96.78	96.35	94.91	0.89	0.690
F/G (g/g)	1.74^a^	1.68^b^	1.70^ab^	0.01	0.030

^a, b^Means within a row followed by different superscript letter differ significantly (P < 0.05).

^1^CON, basal diet; ANT, basal diet supplemented with 50 mg/kg chlorotetracycline; PAL, basal diet supplemented with 10 g/kg palygorskite; SEM, standard error of means (n = 8).

^2^ADG, average daily gain; ADFI, average daily feed intake; F/G, feed to gain ratio.

### Antioxidant-related parameters

The effects of dietary PAL supplementation on the antioxidant status in broilers were presented in Table 3. There were no treatment differences in serum antioxidant parameters of broilers (*P* > 0.05). Compared with CON group, dietary inclusion of PAL reduced the jejunal MDA level in broilers (*P* < 0.05), with its value being similar to that of ANT group (*P* > 0.05). However, dietary treatments did not affect GPX and SOD activities or T-AOC level in jejunum (*P* > 0.05).

**Table 3:** Effects of dietary palygorskite supplementation on the antioxidant status in broilers.

**Items^1, 2^**	**CON**	**ANT**	**PAL**	**SEM**	***P*-value**
**Serum**					
GPX (U/mL)	1,213.87	1,115.48	1,266.02	23.92	0.645
SOD (U/mL)	218.09	231.86	227.34	9.48	0.845
T-AOC (U/mL)	10.50	9.58	10.62	0.37	0.480
MDA (nmol/mL)	3.64	3.63	3.50	0.09	0.807
**Jejunum**					
GPX (U/mg protein)	3.45	4.09	4.20	0.15	0.073
SOD (U/mg protein)	142.08	137.32	142.92	4.51	0.872
T-AOC (U/mg protein)	1.07	1.25	1.08	0.06	0.407
MDA (nmol/mg protein)	0.54^a^	0.42^ab^	0.37^b^	0.03	0.048

^a, b^Means within a row followed by different superscript letter differ significantly (P < 0.05).

^1^CON, basal diet; ANT, basal diet supplemented with 50 mg/kg chlorotetracycline; PAL, basal diet supplemented with 10 g/kg palygorskite; SEM, standard error of means (n = 8).

^2^GPX, glutathione peroxidase; SOD, superoxide dismutase; T-AOC, total antioxidant capacity; MDA, malondialdehyde.

### Immune indices

As presented in Table 4, immune globulin (IgA) and cytokines (TNF-α, IFN-γ and IL-1β) contents were measured to evaluate the effects of PAL supplementation on the immune function of broilers. The administration of PAL reduced concentrations of IFN-γ and IL-1β in serum, and IFN-γ in jejunal mucosa when compared with CON group (*P* < 0.05). Compared to CON group, supplementing the diet with ANT resulted in a lower IL-1β contents in serum and jejunum of broilers (*P* < 0.05), concurrently with their values being comparable between ANT and PAL group (*P* > 0.05). However, PAL treatment did not affect the IgA and TNF-α levels in both serum and jejunum (*P* > 0.05).

**Table 4:** Effects of dietary palygorskite supplementation on the immune function in broilers.

**Items^1, 2^**	**CON**	**ANT**	**PAL**	**SEM**	***P*-value**
**Serum**					
IgA (μg/L)	153.64	168.92	180.41	5.53	0.140
TNF-α (ng/L)	25.75	23.55	23.68	0.45	0.072
IFN-γ (ng/L)	311.11^a^	292.59^ab^	267.05^b^	7.36	0.041
IL-1β (ng/L)	52.55^a^	45.48^b^	44.45^b^	1.37	0.024
**Jejunum**					
SIgA (μg/mg protein)	1.06	1.23	1.24	0.04	0.080
TNF-α (ng/g protein)	5.24	4.98	5.32	0.15	0.628
IFN-γ (ng/g protein)	74.56^b^	64.41^ab^	58.15^b^	2.45	0.014
IL-1β (ng/g protein)	16.26^a^	11.72^b^	14.03^ab^	0.50	0.003

^a, b^Means within a row with different superscripts are different at P < 0.05.

^1^CON, basal diet; ANT, basal diet supplemented with 50 mg/kg chlorotetracycline; PAL, basal diet supplemented with 10 g/kg palygorskite; SEM, standard error of means (n = 8).

^2^IgA, immunoglobulin A; SIgA, secretory immunoglobulin A; TNF-α, tumor necrosis factor-α; IFN-γ, interferon-γ; IL-1β, interleukin-1β.

### Plasma biomarkers and intestinal morphology

Data on the plasma biomarkers of intestinal permeability and mucosal morphology of jejunum in broilers were shown in Table 5. For plasma biomarkers, LPS and D-LA contents were not affected by dietary treatments (*P* > 0.05). Compared with the ANT group, broilers in PAL group exhibited higher value of VH (*P* < 0.05). Moreover, the jejunal VH/CD ratio was also increased by PAL supplementation when compared with the other two groups (*P* < 0.05). However, jejunal CD values were similar among treatments (*P* > 0.05).

**Table 5:** Effects of dietary palygorskite supplementation on the plasma biomarkers of intestinal permeability and mucosal morphology of jejunum in broilers.

**Items^1, 2^**	**CON**	**ANT**	**PAL**	**SEM**	***P*-value**
**Plasma**					
D-LA (mmol/L)	1.43	1.38	1.41	0.06	0.940
LPS (EU/mL)	5.77	5.50	4.98	0.15	0.075
**Jejunum**					
VH (μm)	1,487.56^ab^	1,250.91^b^	1,720.25^a^	79.83	0.048
CD (μm)	217.65	228.77	208.57	9.01	0.676
VH/CD (μm/μm)	6.81^b^	5.59^b^	8.39^a^	0.37	0.004

^a, b^Means within a row with different superscripts are different at P < 0.05.

^1^CON, basal diet; ANT, basal diet supplemented with 50 mg/kg chlorotetracycline; PAL, basal diet supplemented with 10 g/kg palygorskite; SEM, standard error of means (n = 8).

^2^D-LA, D-lactate; LPS, lipopolysaccharide; VH, villus height; CD, crypt depth; VH/CD, villus height to crypt depth ratio.

### Cecal bacterial diversity and community

**Figure 1:**
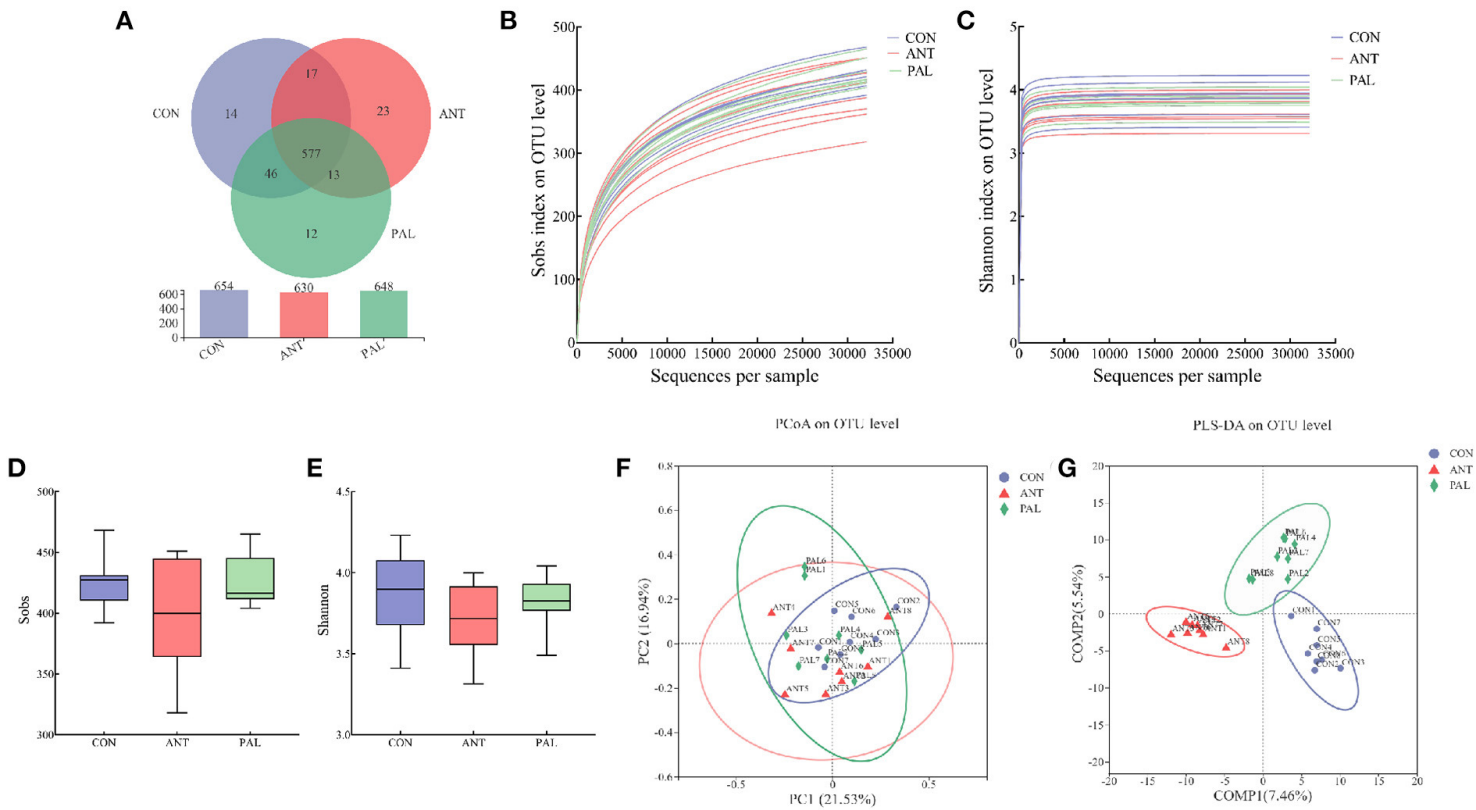
Effects of dietary palygorskite supplementation on the bacterial diversity of cecal content in broilers. **(A)** VENN diagram of bacterial OTU; **(B,C)** rarefaction curves based on observed richness [Sobs, **(A)**] and Shannon diversity index **(B)**; **(D,E)** alpha diversity indexes of Sobs **(D)** and Shannon **(E)**; **(F,G)** principal coordinates analysis [PCoA, **(F)**] and partial least squares discriminant analysis [PLS-DA, **(G)**] on OTU level, and the ellipse represents 95% confidence; CON, basal diet; ANT, basal diet supplemented with 50 mg/kg chlorotetracycline; PAL, basal diet supplemented with 10 g/kg palygorskite.

After sequence quality control, 1,312,520 clean-controlled reads were obtained from 24 cecal samples from three treatments by the high-throughput sequencing. To ensure that samples were representative, data were randomly rarefied to the minimum sequencing depth at 32,116 reads per sample, and clustered into 1,932 OTUs. A total of 14 phyla, 22 classes, 48 orders, 82 families, 167 genera, 320 species, and 702 OTUs were detected in the current study. Based on the Venn analysis of bacteria OTU, there were 577 shared OTUs, and 14, 12 and 23 unique OTUs in CON, PAL and ANT groups, respectively (Figure 1A). The rarefaction curves showed flat trend (Sobs, Figure 1B) or already approached a plateau (Shannon, Figure 1C) as sequence numbers increased, indicating that the rarefied sequencing depths were sufficient to cover the vast majority of bacterial communities in all samples. According to the α-diversity indices, the lowest bacterial richness (Sobs, Figure 1D) and diversity (Shannon, Figure 1E) were in cecal content of broilers fed ANT, although no statistic difference was observed (*P* > 0.05). In addition, the values of Good's coverage ranged from 99.69 to 99.83%, indicating that the majority of the bacterial sequences present in the samples were already procured in this study. As for β-diversity, PCoA of bray-curtis distance at OTU level revealed a less pronounced separation but distinct variation among different groups (Figure 1F). PLS-DA was also introduced as a supervised model analysis, and the score plot successfully showed significant discrepancy in bacterial composition by dietary treatment, although an overlapping cluster was also found between CON and PAL groups (Figure 1G). Moreover, the result from ANOSIM analysis (R > 0 and *P* < 0.05) further demonstrated that samples were significantly separated for the cecal microbiota composition among groups.

**Figure 2:**
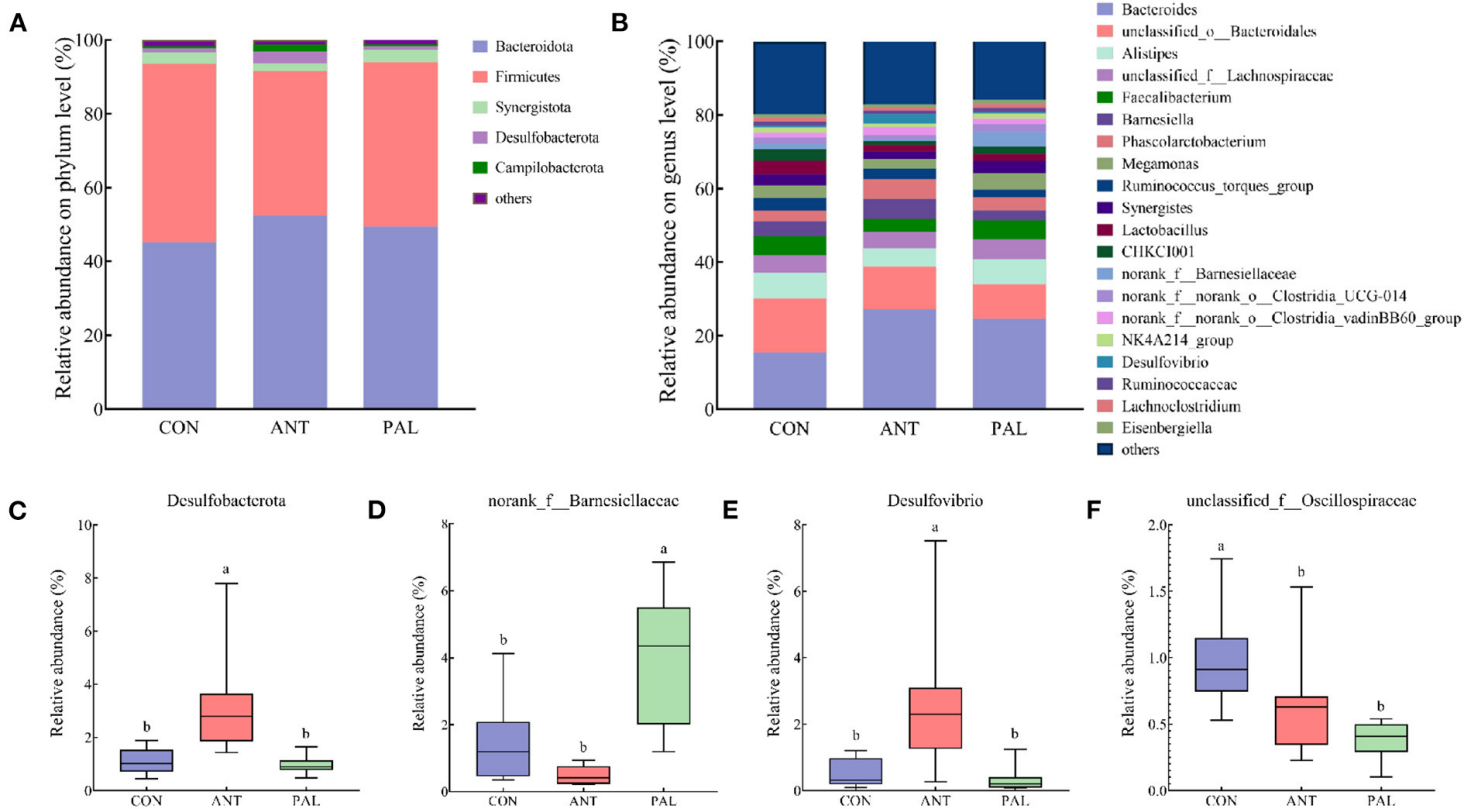
Effects of dietary palygorskite supplementation on the taxonomic composition and distribution of cecal content in broilers. **(A,B)** bar plots of microbial composition at phylum **(A)** and genus **(B)** levels; **(C–F)**, significantly differential bacteria taxa at phylum **(C)** and genus **(D–F)** levels; a,b, different superscripts indicate significant differences among groups (*P* < 0.05); CON, basal diet; ANT, basal diet supplemented with 50 mg/kg chlorotetracycline; PAL, basal diet supplemented with 10 g/kg palygorskite.

Analysis of the cecal microbiota community at phylum and genus levels was shown in Figure 2. The most abundance bacteria at phylum of CON group were discovered to be Firmicutes, while Bacteroidota dominated the microbiota of PAL and ANT groups, and the two most abundant bacterial phyla together accounted for more than 90% of the cecal bacterial community (Figure 2A). These were followed by Synergistota in CON and PAL groups, and Desulfobacterota in ANT group. Moreover, ANT treatment increased the Desulfobacterota abundance in comparison to the CON and PAL groups (Figure 2C, *P* < 0.05). At the genus field, *Bacteroides, unclassified_o__Bacteroidales*, and *Alistipes* were the three most abundant genera across different treatment groups (Figure 2B). Furthermore, *Faecalibacterium* in CON group, *Barnesiella* and *Phascolarctobacterium* in ANT group, and *unclassified_f__Lachnospiraceae* and *Faecalibacterium* in PAL group accounted for relatively high proportions of the cecal microflora (relative abundance > 5%), respectively. Broilers in PAL group had an increased abundance of *norank_f__Barnesiellaceae* (Figure 2D, *P* < 0.05), while, the genus *Desulfovibrio* belonged to phylum Desulfobacterota were higher in ANT group (Figure 2E, *P* < 0.05), when compared with the other two groups, respectively. Additionally, the relative abundance of *unclassified_f__Oscillospiraceae* was significantly decreased in broilers fed with PAL- and ANT- supplemented diets compared with the level in the CON broilers (Figure 2F, *P* < 0.05).

### Correlation of cecal microbiota with growth performance and intestinal health

**Figure 3:**
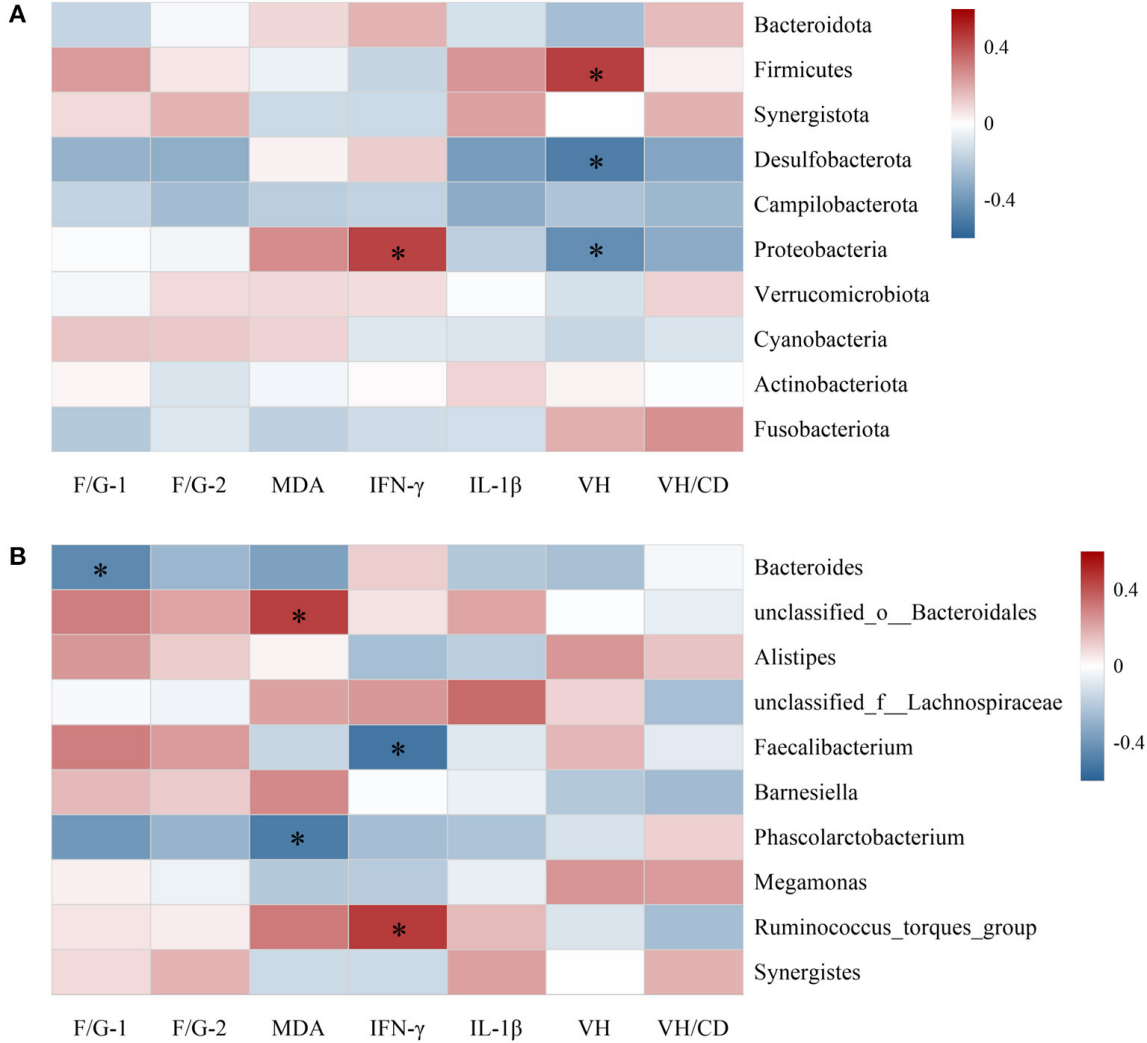
Spearman's correlation analysis of the top 10 abundant cecal microbiota at phylum **(A)** and genus **(B)** levels with growth performance and intestinal health of broilers. Red squares indicate positive correlation, blue squares indicate negative correlation, and significant correlations are marked by * (*P* < 0.05); F/G-1, feed to gain ratio of 22–42 days of age; F/G-2, feed to gain ratio of 1–42 days of age; MDA, IFN-γ and IL-1β, contents of malondialdehyde, interferon-γ and interleukin-1β in jejunal mucosa; VH and VH/CD, villus height and villus height to crypt depth ratio.

Spearman's correlation analysis was conducted to assess the relationship between the top 10 cecal microbiota at phylum (Figure 3A) and genus level (Figure 3B) with growth performance and intestinal health-related parameters of broilers. At phylum level, the average abundances of Proteobacteria and Firmicutes were positively correlated with jejunal IFN-γ content and VH value (*P* < 0.05), respectively, whereas Desulfobacterota were inversely correlated with VH value (*P* < 0.05). At genus field, *Bacteroides* demonstrated negative correlation with F/G during 22–42 days of age (*P* < 0.05). The relative abundances of genera *unclassified_o__Bacteroidales* and *Ruminococcus_torques_group* were positively correlated with jejunal MDA and IFN-γ concentrations, respectively, whereas genera of *Phascolarctobacterium* and *Faecalibacterium* displayed separately negative correlations.

## Discussion

Due to the ban on the AGPs in many countries, there is continuing interest in seeking and developing appropriate and effective alternatives to antibiotics in feed to guarantee animal production and health status. As a non-nutritive and safety additive, dietary clay minerals are proven to improve the productive performance of animals, such as bentonite (Shannon et al., 2017), clinoptilolite (FolnoŽić et al., 2019), PAL (Wang et al., 2019), sericite (Hassaan et al., 2020), etc. Dietary use of clay minerals has been shown to retard the rate of digestive passage through the intestine, and improve nutrient digestion and absorption, thereby resulting a better utilization of feed (Vondruskova et al., 2010). Chalvatzi et al. (2014) reported that the incorporation of PAL decreased the feed conversion ratio of Lohmann Brown hens. Zhang et al. (2013) found that the F/G ratio was lower in piglets after a PAL treatment. These results were in agreement with the superior feed efficiency shown by PAL in this study. However, no significant differences were noted in ADFI and ADG among groups in our research. In contrast, PAL administration was reported to improve the ADFI and ADG of ducks fed diets in pellet form (Wang et al., 2019). Zhang et al. (2017) observed that although PAL inclusion increased ADFI and ADG in broilers, the elevated performance traits could be attributed to the enhanced pellet quality by the addition of PAL. Moreover, the growth performance of broilers and laying hens with mash feed was not affected by the addition of PAL in studies of Qiao et al. (2015) and Cheng et al. (2016, 2018). These inconsistent results may be ascribed to the form of diet, animal species, and the dosage, source and type of PAL. In addition, it was reported that clay minerals were more effective in growth promotion when animals were reared under different stresses (Guo et al., 2017; Chen et al., 2020a). Clay minerals could selectively bind and expel toxic substances and reduce the potential adverse consequences of their action, which can improve the intestinal health and growth performance (Gadde et al., 2017). As broilers grows, the mycotoxins, heavy metals, anti-nutritional factors, and pathogenic organism from feed and environment may accumulated in the gastrointestinal tract, thus affecting broilers' health and growth. Therefore, the positive influences of PAL on feed efficiency during the grower and whole periods may due to the accumulative effect. Moreover, Tang et al. (2014) noted that diet supplemented PAL could take the place of ZnO, an anti-diarrhea compound and growth promoter, to improve growth performance and nutrient digestibility of piglets. In the present study, birds treated with PAL had better feed efficiency with a similar level of that to ANT group. These results indicated that dietary PAL could provide similar benefits as AGPs for growth performance of broilers.

Antioxidant enzymes, such as GPX and SOD, assume crucial roles in detoxifying unwanted free radicals and maintaining normal antioxidant defense system (Wu et al., 2016). T-AOC considers an integrative index to reflect the level of several antioxidant enzymes and non-enzymatic biomolecules with the ability to scavenge free radicals (Wu et al., 2016). MDA, a decomposed product of lipid peroxidation, has been shown as a biomarker for oxidative stress (Cheng et al., 2018). The addition of PAL in the diet has been demonstrated to improve GPX and SOD activities, and thus promote antioxidant capacity in ducks (Wang et al., 2019) and broilers (Chen et al., 2016). Moreover, as reported by Cheng et al. (2018), the benefit effect of PAL was observed to counteract the regressive GPX activity and increased MDA content in broilers fed with lead-contaminated diet, thereby protecting broilers against oxidative stress induced by heavy metal ingestion. In our study, jejunal MDA content was decreased by PAL treatment without significant changes on antioxidant enzymes activities and T-AOC level. Papadopoulos et al. (2016) also demonstrated that oxidative stress and antioxidant mobilization was reduced in sows after a PAL treatment. An *in vitro* study demonstrated that the superior ability of lipid peroxidation inhibition by PAL could be explained by the considerable adsorption and reaction surface sites that stabilize electron deficient species (i.e., hydroxyl radicals) (Cervini-Silva et al., 2015a). Based on these results, the application of PAL in feed could improve antioxidant status and reduce oxidative stress damage in broilers, which may depend on its internal antioxidant property.

Clay minerals are widely employed in feed as immunostimulants to enhance the immune competence and disease resistance of animals (Karimi et al., 2020). IgA, a major serum immunoglobulin and the most abundant antibody synthesized and secreted at mucosal surface, plays main roles in blocking infection and maintaining immune homeostasis (Woof and Kerr, 2006). Furthermore, secretory IgA (SIgA) in the intestinal tract represents the first defense line against the damage to the intestinal epithelium from luminal antigens (e.g., bacteria, viruses, toxins) (Corthésy, 2010). As previously summarized, PAL possesses immunoregulatory property, and the application of PAL would improve immune organ function and promote the antibody production (Zha et al., 2022). Dietary PAL supplementation has shown to increase the intestinal SIgA, IgM and IgG concentrations to enhance the immunity of ducks (Wang et al., 2019). Likewise, Chen et al. (2016) reported that PAL significantly elevated the ileal SIgA level, and tended to increase jejunal IgG content, resulting in improved mucosal immunity in broilers. However, in our study, no significant differences were found in serum IgA and jejunal SIgA in PAL treatment. Proinflammatory cytokines, such as TNF-α, IFN-γ, and IL-1β, can modulate inflammatory reaction to counter infectious agents in gastrointestinal tract, but excessive secretion of these proinflammatory cytokines would induce the endocytosis of tight junction proteins resulting the compromised intestinal integrity (Pietro et al., 2019). Aluminosilicate mineral additive can mitigate the increased proinflammatory cytokine release, relieve inflammatory response, and enhance defensive capacity of immune system in animals suffering immunological challenge (Guo et al., 2017; Chen et al., 2020a). In the current study, dropped IFN-γ concentrations in serum and jejunum and IL-1β level in serum were observed in PAL-treated broilers. PAL, as a classical fibrous aluminosilicate mineral, has been reported to exhibit an anti-inflammatory characteristic, as demonstrated by inhibitions of edema and neutrophils migration, and reduction in gene expression levels of pro-inflammatory cytokines in murine inflammation model (Cervini-Silva et al., 2015b; López-Pacheco et al., 2017). This finding is partially in agreement with result of Chen et al. (2020a), who reported that the administration of PAL down-regulated the mRNA expression levels of intestinal TLR4 and IFN-γ of broilers, indicating that PAL may restrain the synthesis of proinflammatory cytokines *via* modulating the signaling pathway of TLR4/MyD88/NF-κB. In extension, Zhang et al. (2013) reported that the positive immune system response of supplementing PAL may be correlated with the enriched intraepithelial lymphocytes in intestine. Besides, jejunal IL-1β content was also depressed by dietary PAL, with the value being similar to that in ANT group. These results further showed a promise for PAL as an antibiotic alternative owing to its efficacy in enhancing immunological function.

The intestinal physical barrier plays a vital role in the resistance to enteric pathogens invasion and nutrient absorption (Chen et al., 2019). D-LA is a metabolite of bacterial fermentation of carbohydrates, and LPS is a composition of cell wall in gram-negative bacteria (Zhang et al., 2013). Under normal physiological circumstance, D-LA or LPS cannot translocate from the epithelial mucosa and into circulation, and elevated D-LA and LPS levels in plasma are indicative of raised permeability and ruined integrity of intestine (Pietro et al., 2019). Chen et al. (2020a) reported that dietary PAL exerted a protective effect on intestinal integrity in LPS-challenged broilers, as evident by a decreased circulating D-LA concentration coincided with downregulation of mucosal mRNA expression level of tight junction proteins. In addition, dietary PAL supplementation reduced serum DAO activity, an another important index reflecting intestinal permeability, in broilers at 21 days of age (Chen et al., 2016). However, in our current study, supplementation of PAL to broilers had no significant effects on plasma D-LA and LPS contents. Possible reason that could explain the discrepant results is that the gut barrier function is comparatively mature and less susceptible to damage in elderly broiler (Zhang et al., 2013). Micromorphological features, including VH, CD, and VH/CD, are important indicators of the digestive and absorptive capacity in intestine tract (Chen et al., 2019). In this study, the inclusion of PAL in broiler diet increased values of VH and VH/CD in jejunum. The longer villus has a mature and active functional epithelium, and provides a bigger surface area to absorb nutrient (Wang et al., 2021b). The effect of PAL in stimulating intestinal morphology was consistent with previous reported results (Zhang et al., 2013; Chen et al., 2016; Tzora et al., 2017). Aluminosilicates could absorb or detoxify toxins, bacteria and viruses, as well as take up harmful gases in gastrointestinal tract due to their unique structure and high ion-exchange capacity (Qiao et al., 2015; Du et al., 2019). On the other hand, the huge specific surface area of PAL allows it to form a protective screen by adhering to the mucous membrane, and thus diminish irritations of hazardous substances to intestinal physical barrier (Kotsampasi et al., 2017). Moreover, based on aforementioned mechanisms, dietary PAL would improve intestinal morphological characteristics through the promotion of mucosal antioxidant status and immunity, or the relief from oxidative stress and inflammatory response. A similar study conducted in broilers revealed that the better mucosal morphology by montmorillonite contributed to the increased nutrient digestibility as well as intestinal defense function, which may be relate to the improvement of the intestinal microecological environment (Liu H. et al., 2020). Besides, the shorter villi in birds fed ANT diet may also be correlated with the alteration of bacteria populations (Baurhoo et al., 2007). Overall, it may be speculated that dietary PAL may be more beneficial than antibiotics in maintaining the intestinal barrier function.

The intestinal microbiota occupies indispensable part in the intestinal morphology, barrier function, and nutrition metabolic, particularly in the cecum, where is the key site of the bacterial fermentation in the gut. And it is generally agreed that a stable intestinal microflora is highly connected to the improvement in growth performance and host health. Jin et al. (2021) demonstrated that feed amendment with PAL brought a greater bacterial diversity in the cecal contents from Partridge Shank chickens, which was supported by the increased values of Chao1 estimator and Sobs. In contrast, Chalvatzi et al. (2016) found that there was a higher intestinal microbial homogeneity in laying pullets among the PAL individuals compared with controls. However, the findings from Liu H. et al. (2020) showed that dietary supplementation with montmorillonite has no impact on the α-diversity of cecal bacteria on weaned piglets. Consistently, in this present study, PAL did not result in an alteration in microbial community diversity and richness compared to the control feed group. Since the vast diversity of gut microbiota, variations of animal strain, diet type, growth stage, and stocking density may explain the observed differences (Wu et al., 2019). According to β-diversity analysis of PCoA, PLS-DA and ANOMIS, PAL treatment significantly altered the cecal microbiota structure of broilers. This change, combined with results of microflora abundances, may be related with the shift of community composition by diet treatment rather than a change in overall microbial flora diversity.

Regardless of treatment, the cecal microbial community was predominantly comprised by phyla Bacteroidota and Firmicutes, which were consistent with previous researches conducted in broilers (Jin et al., 2021; Qiao et al., 2021). The proportion of phylum Desulfobacterota, as well as genus Desulfovibrio, was enhanced in ANT groups. Desulfovibrio, known as sulfate-reducing bacteria, can inhibit the enzyme involved in nicotinamide adenine dinucleotide recycling, and ultimately remove hydrogen that restricted the production of short-chain fatty acids (SCFAs) (Li et al., 2016). Moreover, members of Desulfovibrio were beneficial to modulate microbial community within the cecal ecosystem, and improve energy recovery from digestive tract chyme (Li et al., 2016). Hence, the increased amount of genus *Desulfovibrio*, also reflected at Desulfobacterota phylum level, may participate the improvement of growth performance in broilers fed ANT. Clay minerals can selectively to bind or agglutinate to certain bacterium, and differentially affect bacterial survival *in vitro* (Brennan et al., 2014). Moreover, in-feed supplementation of clay minerals also was believed to modulate the growth of specific bacteria groups within gastrointestinal tract (Bederska-Łojewska and Pieszka, 2019; Liu H. et al., 2020; Jin et al., 2021). Here, With the presence of PAL, broiler chicks had higher abundance of genus *norank_f__Barnesiellaceae*, belonging to family *Barnesiellaceae* which is SCFA-producing bacteria and gut-friendly probiotic. As proposed by Chen et al. (2022), intestinal *norank_f__Barnesiellaceae* was positively related with antioxidant capacity and displayed an opposite correlation with inflammatory response in the common carp, which may have a synergistic effect on growth performance. These evidences was in good agreement with our above-mentioned results that dietary PAL showed a more pronounce effect in immunological function than that of ANT group. Moreover, excessive abundance of family *Oscillospiracea* has been shown to induce inflammation and link to sub-health status in host (Wang et al., 2021a). Therefore, the suppression of cecal *unclassified_f__Oscillospiraceae* may partly explain the immune modulation by PAL supplementation.

The heatmaps of spearman's correlation have disclosed the tightly linked interaction between the cecal microbiota and parameters relating the growth performance (feed conversion efficiency) and intestinal health (antioxidant status, immune function and mucosal morphology), which is consistent with our supposition. *Bacteroides* is one of the most predominant genera in the cecal microbiota of broilers. Members of *Bacteroides* have extensive capabilities for plant-derived carbohydrates metabolism and are positively correlated with growth performance of their hosts (Yan et al., 2019). Yun et al. (2021) reported that genus *Bacteroides* could improve nutrient digestion and absorption of the host through carbohydrate metabolism, and broilers with high feed efficiency exhibited a higher abundance of *Bacteroides* which was in line with our finding. As SCFA-producers, both *Phascolarctobacterium* and *Faecalibacterium* have been confirmed to reduce intestinal permeability, improve the gut barrier function, and thus be beneficial for host health (Luo et al., 2019). The increased proportion of *Phascolarctobacterium* has been reported to be associated with the improvements of the antioxidant capacity and lipid metabolism (Jiang et al., 2019). Tsao et al. (2021) stated that species of *Faecalibacterium* exerted anti-inflammatory activity in animal gut, and this may be related with the decreased levels of pro-inflammatory cytokines found in PAL-supplemented broilers. Despite literature indicated that members of order *Bacteroidales* were involved in the gut dysfunction by the degradation of mucin, limited data was known about the relationship between genus *unclassified_o__Bacteroidales* and intestinal antioxidant status (Zitomersky et al., 2013). In the present study, *unclassified_o__Bacteroidales* was positively correlated with jejunal MDA level, implying that this genus might be a possible symbol of the level of intestinal oxidative stress in broilers. Moreover, phylum Proteobacteria and genus *Ruminococcus_torques_group* were found positively associated with the jejunal IFN-γ content. These species, which contain several pathogenic bacteria, can damage the mucosal barrier and facilitate intestinal inflammation (Zitomersky et al., 2013; Minicis et al., 2014). In our study, the results of VH value and phylum Desulfobacterota proportion were contrary in broilers of ANT groups. As mentioned above, the changed mucosal morphology by dietary ANT was associated with the alteration of intestinal microflora (Baurhoo et al., 2007). Liu Z. et al. (2020) reported that broilers supplemented with sanguinarine achieved higher VH value and Firmicutes relative abundance. Additionally, mucosal morphology was improved, but relative abundance of Proteobacteria was decreased in ileum of piglets after supplementing clay mineral (Liu H. et al., 2020). These previous reports were in line with our finding. Overall, dietary supplementing of PAL played a positive role in regulating gut microflora, which had the potential relationship with promoted growth performance and intestinal health, but the underlying mechanism warrants further investigation exploration.

## Conclusion

Collectively, this investigation provided reliable data that dietary supplementation with PAL had similar or better effects in improving growth performance, oxidative status, immunity and intestinal barrier function, and regulating cecal microbiota, when compared with antibiotic inclusion. Moreover, the alteration of intestinal microbiota by supplementation with PAL might be associated with beneficial effects on growth performance and intestinal health of broilers. All this suggested that PAL can be used in antibiotic-free diet to maximize growth performance and protect intestinal health in animals.

## Data availability statement

The datasets presented in this study can be found in online repositories. The names of the repository/repositories and accession number(s) can be found at: https://www.ncbi.nlm.nih.gov/PRJNA848965.

## Ethics statement

The animal study was reviewed and approved by Nanjing Agricultural University Institutional Animal Care and Use Committee.

## Author contributions

MD and YZ conceived and designed the experiment. MD, SW, and HZ performed the animal experiment and sample analysis. MD, YC, and CW analyzed data and authored the final article. YZ supervised and provided continuous guidance for the experiments. All authors contributed to the article and approved the final manuscript.

## Funding

The study was financially supported by the Nation Natural Science Foundation of China (No. 31872405).

## Conflict of interest

The authors declare that the research was conducted in the absence of any commercial or financial relationships that could be construed as a potential conflict of interest.

## Publisher's note

All claims expressed in this article are solely those of the authors and do not necessarily represent those of their affiliated organizations, or those of the publisher, the editors and the reviewers. Any product that may be evaluated in this article, or claim that may be made by its manufacturer, is not guaranteed or endorsed by the publisher.
